# Female donor gender is associated with a decrease in liver transplant survival of male recipients independent of donor and recipient anthropometrics

**DOI:** 10.1590/0102-67202025000024e1893

**Published:** 2025-10-27

**Authors:** Marcio Fernandes CHEDID, Lucas PREDIGER, Gabriel LAZZAROTTO-DA-SILVA, Jane CRONST, Alexandre DE ARAUJO, Tomaz de Jesus Maria GREZZANA, Luciano Zubaran GOLDANI

**Affiliations:** 1 Universidade Federal do Rio Grande do Sul, Hospital de Clínicas de Porto Alegre, Liver Transplant Program – Porto Alegre (RS), Brazil.; 2 Universidade Federal do Rio Grande do Sul, Faculty of Medicine, Medical Sciences, Postgraduate Program in Medicine – Porto Alegre (RS), Brazil.

**Keywords:** Liver Transplantation, Neoplasms, Chemotactic Factors, Survival, Infections, Transplante de Fígado, Neoplasias, Fatores Quimiotáticos, Sobrevida, Infecções

## Abstract

**Background:**

Data on the influence of donor gender on post-liver transplant outcomes is scarce and is lacking.

**Aims:**

The aim of this study was to evaluate the prognostic factors of mortality in patients undergoing liver transplantation (LT) with a thorough evaluation of the influence of the donor variables.

**Methods:**

All patients undergoing LT at a single center from December 2011 to December 2018 were included. The main outcome measure of the study was overall patient survival. The mortality predictors were evaluated using Cox regression.

**Results:**

The study analyzed 202 patients, 118 (58.1%) being males, and the average age was 54.19±11.66 years. Post-LT survival for the entire cohort of 202 patients as assessed by the KaplanMeier method at 1, 3, 5, and 7 years was 81.6, 73.1, 67.6, and 63%, respectively. The only predictor of increased overall mortality was female donor gender [HR 1.918, 95%CI 1.150–3.201, p=0.013]. Weight and height differences between donor and recipient were not related to mortality (p=0.545 for weight and p=0.964 height).

**Conclusions:**

Female donor gender was associated with an increase in overall post-LT mortality, especially for male recipients, regardless of anthropometric parameters. For male patients receiving livers from female donors, infection was the most common cause of mortality, occurring in the first year following LT.

## ARTICLE HIGHLIGHTS

Post-liver transplant survival has achieved little improvement over the last decade.

Data on the influence of donor gender on post-liver transplant outcomes is scarce.

Analyzing a cohort of 202 consecutive patients receiving a liver transplant, female donor gender was the only predictor of increased overall mortality [HR 1.918, 95%CI 1.150–3.201, p=0.013].

The reason for those inferior outcomes related to LT employing livers from female donors was not related to anthropometric differences.

## Summary

**CENTRAL MESSAGE:**

Post-liver transplant survival has achieved little improvement over the last decade. Several prognostic factors of mortality after liver transplant have been identified, including donor age, long cold ischemia time, long warm ischemia time, moderate hepatic steatosis in the donor, and others. However, data on the influence of donor gender on post-liver transplant outcomes is scarce.

**PERSPECTIVES:**

The female donor gender was associated with an increase in overall post-liver transplant mortality, especially for male recipients. The reason for those inferior outcomes of males receiving livers from female donors was not related to anthropometric differences. For male patients receiving livers from female donors, infection was the most common cause of mortality occurring in the first year following LT. Future studies are needed to elucidate whether immune discrepancies would be accountable for increased mortality of males receiving livers from female donors.

## VISUAL ABSTRACT

**Figure:**
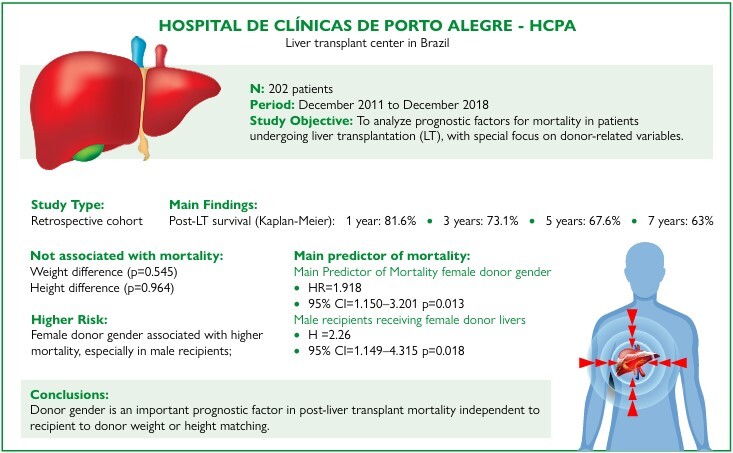

## RESUMO

**Racional:**

Os dados sobre a influência do sexo do doador nos resultados pós-transplante de fígado são escassos ou faltam.

**Objetivos:**

Analisar os fatores prognósticos de mortalidade em pacientes submetidos a transplante hepático (TH) mediante avaliação minuciosa da influência das variáveis do doador.

**Métodos:**

Foram incluídos todos os pacientes submetidos a TH em um único centro no período de dezembro de 2011 a dezembro de 2018. O principal desfecho do estudo foi a sobrevida global do paciente. Os preditores de mortalidade foram avaliados por meio da regressão de Cox.

**Resultados:**

Foram analisados 202 pacientes, sendo 118 (58,1%) do sexo masculino, e a idade média foi de 54,19±11,66 anos. A sobrevida pós-TH para toda a coorte de 202 pacientes avaliada pelo método Kaplan-Meier em 1, 3, 5 e 7 anos foi de 81,6, 73,1, 67,6 e 63%, respectivamente. O único preditor de aumento da mortalidade geral foi o sexo do doador feminino [HR 1,918, IC95% 1,150–3,201, p=0,013]. As diferenças de peso e altura entre doador e receptor não foram relacionadas à mortalidade (p=0,545 para peso e p=0,964 para altura).

**Conclusões:**

O sexo feminino do doador foi associado a um aumento na mortalidade global pós-TH, especialmente para receptores do sexo masculino, independentemente dos parâmetros antropométricos. Para pacientes do sexo masculino que receberam fígados de doadores do sexo feminino, a infecção foi a causa mais comum de mortalidade ocorrida no primeiro ano após o TH.

## INTRODUCTION

 Post-liver transplant survival has achieved little improvement over the last decade^22^. Several prognostic factors of mortality after LT have been identified, including donor age, long cold ischemia time, long warm ischemia time, moderate hepatic steatosis in the donor, and others^1,6,7,9,10^. 

 Data on the influence of donor gender on post-liver trans plant outcomes is scarce. The objective of this study was to iden tify the prognostic factors of mortality in patients undergoing LT. 

## METHODS

 All adult patients who underwent a first LT at the authors’ institution from December 2011 to December 2018 were included. Pediatric patients (under 18 years of age), partial graft recipients (split livers and partial grafts from living donors), and patients with concurrent transplants (combined liver and kidney transplant) were not included in this analysis. 

 This study was approved by the Institution Ethics Committee (number 2017-0271). The study researchers signed a confidentiality agreement regarding the use of the collected data. Informed consent was obtained from all patients who were alive by the time of the study start. 

 All transplants were performed using the "piggyback" technique. Generally, the threshold for performing blood autotransfusion using blood collected by the intraoperative blood recovery device for autotransfusion was 1,000 mL of bleeding (or hemodynamic instability occurrence related to hypovolemia)^21^. Fresh frozen plasma, cryoprecipitate, and platelets were administered as needed according to thromboelastographic evaluation. 

 Abdominal Doppler ultrasound was performed routinely to screen for hepatic vascular complications^5,11^. Oral feeding was started in the intensive care unit shortly after extubation. Immunosuppression was based on the use of tacrolimus, mycophenolate, and steroids. 

 The study’s primary endpoint was overall post-LT patient survival. The patients were followed until their death or until the end of the study period. Post-LT infectious episodes also were studied and characterized. There were no follow-up losses during the study period. 

 The following variables collected in the immediate pretransplant period were analyzed: recipient age, gender, ethnicity/race, height, weight, body mass index (BMI), presence of diabetes mellitus (DM), MELD (model for end-stage liver disease) score, hepatitis C virus (HCV) infection, presence of hepatocellular carcinoma (HCC), infection prior to LT, ascites prior to LT, dialysis prior to LT, total bilirubin (TB), international normalized prothrombin time ratio (INR), albumin, sodium, creatinine, platelets, albumin-bilirubin score (ALBI)^2^, donor gender, donor age, donor weight, donor height, recipient weight, and recipient height. Data from laboratory tests were collected up to 15 days before LT. Demographic and laboratory data of the donors were also collected and analyzed. 

 For the identification of the prognostic factors related to each of mortality, univariate analysis was performed using the Cox regression method using the variables described above. Variables whose p-value was <0.05 in each of the univariate analyses for the outcomes were included in multivariable Cox regressions respective to each outcome. For all analyses, p-values<0.05 were considered statistically significant. Analyses were performed using SPSS V.18 software (IBM Corporation, Armonk, New York, U.S.). 

## RESULTS

 A total of 202 patients who underwent a first LT were included (Table 1), of which 118 (58.4%) were male, and the remaining 84 patients (41.6%) were female. The mean age was 54.19±11.66. The predominant ethnicity was Caucasian (94.7% of patients). Notably, 86 patients (28.6%) had diabetes mellitus. The main cause of end-stage liver disease was HCV infection, accounting for 125 cases (61.9%). Additionally, HCC in the setting of cirrhosis occurred in 115 patients (56.9%), being the main indication of LT in this cohort. The calculated median MELD score was 13 (interquartile range — IQR=10–19). 

**Table 1:** Demographic variables of 202 consecutive patients who underwent liver transplantation at a single center.

	n (%)	Mean±DP	Median+IQR
Age		54.19±11.66	57 (IQR 49.75–62)
Male gender	n=118 (58.4%)		
Body mass index		27.76±5.457	27 (IQR 24–31)
MELD score		16±9	13 (IQR 10–19)
MELD-Na score		16±8	13 (IQR 10–19)
HCV infection	n=125 (61.9%)		
Hepatocellular carcinoma	n=115 (56.9%)		
Albumin		3.18±0.73	3 (IQR 3–3.5)
Bilirubin		4.51±8.4	1,70 (IQR 0.9–3.22)
INR		1. 77±1,58	1,37 (IQR 1.21–1.68)
Sodium		139.66±4.13	
Creatinine		1.04±0.84	0,8 (IQR 0.66–1.07)
Diabetes	n=86 (28.3%)		

DP: standard deviation; IQR: interquartile range; MELD: model for end-stage liver disease; MELD-Na: model for end-stage liver disease-sodium; HCV: hepatitis C virus; INR: International Normalized prothrombin time Ratio.

**Figure 1:**
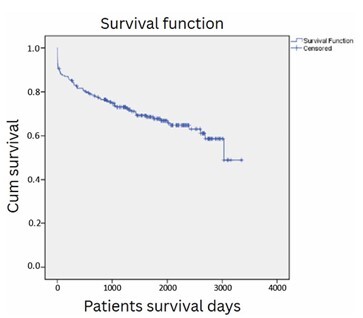
Survival analysis of 202 consecutive patients who underwent liver transplantation at a single center.

 Post-LT survival for the entire cohort of 202 patients as assessed by the Kaplan-Meier method at 1, 3, 5, and 7 years was 81.6, 73.1, 67.6, and 63%, respectively (Figure 1). There were 18 deaths during the first 30 days post-LT days (30-day mortality of 8.9%). Between the 31st and 180th days, there were 7 deaths. Between the 181st and 365th day post-LT, 13 other deaths occurred. Overall, a total of 38 deaths occurred during the first post-LT year for the 202 transplant patients, resulting in a 1-year actual survival of 81.1%. 

 During the first 30 post-LT days, the main cause of death was graft dysfunction (n=12) (either primary or secondary to vascular thrombosis or large-for-size grafts). Among the patients who died during the first 30 post-LT days, 3 suffered from hepatic artery thrombose, 2 from portal vein thrombose, and 4 from hemorrhagic shock. There were three abdominal compartment syndromes caused by large-for-size liver grafts. During this period, 5 of the 18 deaths (27.78%) were due to infections. 

 From the 31st to 180th day post-LT, the main cause of death was infection (six of the seven deaths were caused by infection). Between days 181 and 365 post-LT, infection was also the main cause of death (8 of the 13 deaths in this period were caused by infection). 

Table 2 presents the results of univariate analysis by Cox regression with the outcome of mortality occurring throughout the study follow-up. According to this analysis, female donor gender (HR 1.806, 95%CI 1.091–2.988; p=0.021, p<0.05) was the only predictor of overall post-LT mortality. 

**Table 2:** Univariate analysis of the factors to the outcome of overall death (entire post-transplant follow-up) of 202 consecutive patients who underwent liver transplantation at a single center.

	Hazard ratio [95%CI]	p-value
Age	0.996 [0.976–1.016]	0.677
Female gender	0.830 [0.511–1.348]	0.451
Receptor height	1.002 [0.978–1.026]	0.884
Receptor weight	1.006 [0.990–1.023]	0.468
Body mass index	1.026 [0.973–1.080]	0.342
Diabetes	1.367 [0.836–2.237]	0.213
MELD score	1.003 [0.977–1.031]	0.811
MELD-Na score	1.003 [0.977–1.030]	0.840
HCV infection	0.912 [0.563–1.477]	0.708
Hepatocellular carcinoma	1.003 [0.623–1.614]	0.991
Total bilirubin	1.011 [0.986–1.037]	0.389
INR	0.886 [0.702–1.118]	0.309
Sodium	1.008 [0.951–1.068]	0.788
Creatinine	0.828 [0.573–1.196]	0.314
Platelets	1.022 [0.960–1.088]	0.501
Albumin	0.749 [0.546–1.027]	0.072
Albumin-bilirubin grade (ALBI) 2 — categories Grade 1/grade 2 (=-1.39) Grade 3 (>-1.39)	1.155 [0.668–1.995]	0.606
Pre-transplant dialisis	1.124 [0.486–2.598]	0.785
Infection prior to LT	1.301 [0.744–2.275]	0.355
Ascites prior to LT	1.405 [0.876–2.253]	0.158
Donor infection	0.956 [0.550–1.644]	0.875
Donor age	1.011 [0.996–1.027]	0.154
Donor gender, female	1.806 [1.091–2.988]	0.021
Donor BMI	0.988 [0.914–1.069]	0.771
Donor height	0.696 [0.037–13.192]	0.809
Donor weight	0.996 [0.976–1.016]	0.669
Height difference between donor and recipient	1.000 [0.980–1.021]	0.964
Weight difference between donor and recipient	0.996 [0.982–1.010]	0.545
Infection, receptor during transplant hospitalization	0.793 [0.468–1.345]	0.390
Infection during first 90 post-transplant days	0.646 [0.403–1.035]	0.069
Infection during first year	0.689 [0.430–1.104]	0.121

CI: confidence interval; MELD: model for end-stage liver disease; MELD-Na: model for end-stage liver disease-sodium; HCV: hepatitis C virus; INR: International Normalized prothrombin time ratio; LT: liver transplant; BMI: body mass index.

 Anthropometric parameters of donor and recipient also were studied. The difference between donor and recipient weight was not related to a decreased post-LT survival (p=0.545, p>0.05). Likewise, the difference between donor and recipient height was not related to a decreased post-LT survival (p=0.964, p>0.05). 

**Figure 2:**
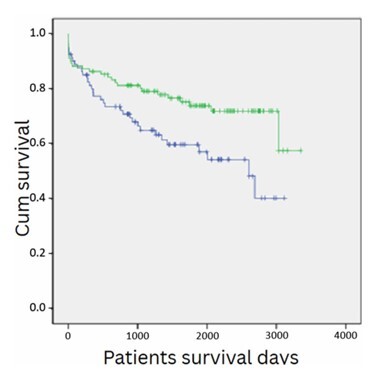
Survival analysis of 202 consecutive patients who underwent liver transplantation at a single center, stratifying by donor gender, depicting a lower survival for patients who received a liver graft from a female donor (p=0.013).

Figure 2 shows the analysis of post-transplant survival using the Kaplan-Meier method, stratified by donor gender. Post-LT survival for patients who received a liver graft from a male donor was 86.3, 79.1, 73.8 and 71.8% at 1, 3, 5, and 7 years, respectively, *versus* 77.4, 64.9, 59.6, and 54.2% for patients who received a liver from a female donor (p=0.013, p<0.05). 

**Figure 3:**
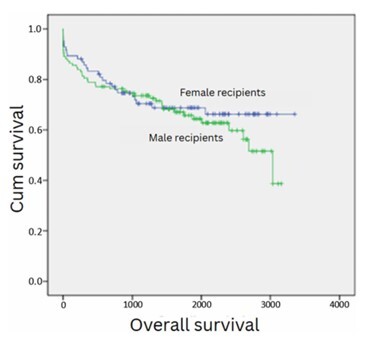
Survival analysis of 202 consecutive patients who underwent liver transplantation at a single center, stratifying by recipient gender, depicting a similar survival (p=0.45).

Figure 3 depicts the analysis of post-transplant survival stratified by recipient gender. Post-LT survival for male recipients was not different from that of female recipients (p=0.45, p>0.05). 

**Figure 4:**
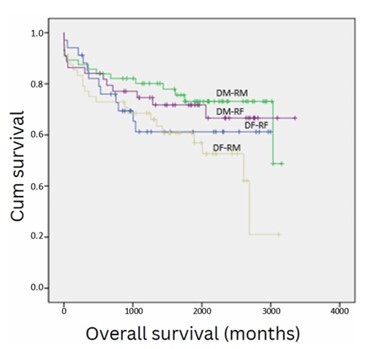
Survival analysis of 202 consecutive patients who underwent liver transplantation at a single center, stratifying in four groups by donor and recipient gender. DM: donor male; DF: donor female; RM: recipient male; RF: recipient female.

Figure 4 shows recipient survival stratified in four groups by donor and recipient gender. The highest survival occurred for male patients receiving a liver from a male donor (1 year=85.7%, 3 years=80.1%, 5 years=73.1%, and 7 years=73.1%), whereas the lowest survival occurred for male patients receiving a liver graft from female donors (1 year=75%, 3 years=68.5%, 5 years=60.8%, and 7 years=52.6%) (p=0.028, p<0.05). 

 Analyzing only male gender recipients, receiving a liver from a female gender donor as compared with receiving a liver from a male donor was associated with an increase of 2.26 times in overall post-LT mortality (95%CI 1.149–4.315, p=0.018, p<0.05). 

 No statistically significant survival difference was detected for the comparisons between all the other donor-recipient gender comparisons (female donor to female recipient *vs.* male donor to male recipient, p=0.213, p>0.05), (female donor to female recipient *vs.* female donor to male recipient, p=0.466, p>0.05, female donor to female recipient *vs.* male donor to female recipient, p=0.513, p>0.05, male donor to male recipient *vs.* male donor to female recipient, p=0.689, p>0.05, female donor to male recipient *vs.* male donor to female recipient, p=0.12, p>0.05). 

 Additional analyses were performed in the group that had the lowest overall survival (male recipients receiving livers from female donors). Among that subgroup of patients, the difference in donor and recipient weight was not related to a decreased post-LT survival (p=0.425, p>0.05). Likewise, the difference between donor and recipient height was not related to a decreased post-LT survival (p=0.114, p>0.05). 

 As an attempt to quantify the weight mismatches between donor and recipients, we calculated the difference between the weight of the donor and that of the recipient (donor weight — recipient). Thus, we have grouped the donor-to-recipient weight differences into three groups. The first group included individuals with donor-recipient weight differences of more than 10 kg (the recipient weighed 10 or more kilos than the donor); for the second group donor-recipient, weight difference was between -10 and +10 kg; the third group had a donor-recipient weight difference of more than 10 kg (the recipient weighed 10 or less kilos than the donor). Post-LT survival for the three groups was not statistically different (p=0.399, p>0.05). 

 In order to quantify the height mismatches between donor and recipients, we calculated the difference between the height of the donor and that of the recipient (donor height — recipient height). For quantifying the height differences between the donor and the recipient, we also grouped the height differences into three groups. The first group encompassed individuals with donor-recipient height differences of less than 10 cm (the recipient was 10 or more centimeters taller than the donor); for the second group, donor-recipient height differences were between -10 and +10 cm; the third group had patients with donor weight-recipient height differences of 10 or more kilograms (the recipient was 10 or more centimeters shorter than the donor). Post-LT survival for the three groups was not statistically different (p=0.772, p>0.05). 

 Among the group of 46 male patients receiving livers from female donors, 12 deaths occurred during the first year (mortality rate of 26.1%) as compared to a 14% 1-year mortality rate of male recipients who received livers from male donors. The causes of death of the 12 male recipients who received livers from female donors included infection (n=7, 58.3%), hemorrhagic shock (n=1), hepatic artery thrombosis (n=1), portal vein thrombosis (n=1), and unknown (n=2). In contrast, in the group of male patients who received liver allografts from male donors, only 12.5% of the deaths occurring during the first post-LT year were caused by infection. 

## DISCUSSION

 In the present study, the outcomes of 202 consecutive adult patients undergoing LT at a single center are analyzed. The main cause of end-stage liver disease in this cohort was cirrhosis secondary to HCV infection, which occurred in 125 out of 202 cases (61.9%). The main indication for LT was HCV cirrhosis with or without HCC. Post-LT survival at 1, 3, 5, and 7 years was comparable to international parameters^15,24^, being comparable to that of other international centers. 

 Among all parameters studied, female donor gender was the only predictor of post-LT mortality, being associated with an increase of 81.6% in overall post-LT mortality. Remarkably, the association of female donor gender to post-LT recipient mortality was detected in both male and female recipients. Moreover, this association of female donor gender and mortality was stronger for male recipients. Thus, male recipients who received LT from female donors experienced a 2.2 times increase in overall mortality as compared to male recipients who received livers from male donors (12% higher mortality at 5 years and 21% higher at 7 years post-LT). 

 Notably, neither weight nor height incompatibilities were related to inferior outcomes in this cohort. Thus, this is the first study showing that the deleterious association of female liver donors-male recipients is not related either to weight or to height incompatibility between donor and recipient. 

 The few available literature studies on the subject exhibit conflicting results^3,4,8,12,13,16,20,23,25^. While some studies^4,16,20,25^ revealed that livers from female donors pose an increased risk of death, other studies do not share the same results^3,18^. A recent systematic review and meta-analysis showed that female donors in male recipients was associated with an 83% decrease in post-LT survival^19^. 

 To date, the reason for this inferior survival for males receiving livers from female donors has not been elucidated. Although it could be related to different sizes among human males and females (a small-for-size effect), the present study did not detect any association of weight and height mismatch to a decrease in overall post-LT survival. Moreover, even in the group of male recipients who received a liver from female donors, neither weight nor height differences accounted for any increase in mortality. 

 As an attempt to elucidate the reasons for the death of males receiving livers from female donors, a search for the reasons for graft failures was carried out. Interestingly, 58% of all first-year mortality in this group was caused by infection. In contrast, infection was responsible for only 12.5% of all first-year deaths of males who received livers from male donors. 

 Post-transplant infection was not a predictor of mortality in this study. However, infection was associated with a significant increase in post-transplant hospital stay. It is possible that greater rigor in immunosuppression could prevent the occurrence of infection in patients undergoing LT, positively impacting the long-term survival. The most common site for infection in our series was sepsis without a defined infectious site, with a total of 20 episodes (31.8%). The abdomen, the most common site of infection in literature studies, was only the third most common site of infection in the present study (20.6% of cases). These data differ in relation to two recent review articles, which establish the abdomen as the most common site of infection in LT recipients^14,17^. 

 The present study has some limitations. The main one is the retrospective design. The nature of retrospective studies increases the chance of measurement biases. The absence of randomization is another limitation. However, it would be impossible to randomize LT patients to receive a liver graft from a specific deceased donor gender. 

## CONCLUSIONS

 In summary, female donor gender was associated with an increase in overall post-LT mortality, especially for male recipients. The reason for those inferior outcomes of males receiving livers from female donors was not related to anthropometric differences. For male patients receiving livers from female donors, infection was the most common cause of mortality occurring in the first year following LT. Thus, future studies are needed to elucidate whether immune discrepancies would be accountable for the increased mortality of males receiving livers from female donors. 

## Data Availability

The information regarding the investigation, methodology, and data analysis of the article is archived under the responsibility of the authors.
